# Application of Monoclonal Antibodies Developed Against the IpaJ Protein for Detection of Chickens Infected With *Salmonella enterica* Serovar Pullorum Using Competitive ELISA

**DOI:** 10.3389/fvets.2019.00386

**Published:** 2019-11-05

**Authors:** Kequan Yin, Jingwei Ren, Yue Zhu, Lijuan Xu, Chao Yin, Yang Li, Yu Yuan, Qiuchun Li, Xinan Jiao

**Affiliations:** ^1^Key Laboratory of Prevention and Control of Biological Hazard Factors (Animal Origin) for Agri-food Safety and Quality, Ministry of Agriculture of China, Yangzhou University, Yangzhou, China; ^2^Jiangsu Key Lab of Zoonosis/Jiangsu Co-innovation Center for Prevention and Control of Important Animal Infectious Diseases and Zoonoses, Yangzhou University, Yangzhou, China; ^3^Joint International Research Laboratory of Agriculture and Agri-Product Safety of Ministry of Education of China, Yangzhou University, Yangzhou, China

**Keywords:** *Salmonella enterica* serovar Pullorum (*S*. Pullorum), monoclonal antibody (MAb), competitive ELISA, IpaJ, plate agglutination test (PAT)

## Abstract

Pullorum disease remains an epidemic in the poultry industry in China. The causing pathogen is a host-restricted *Salmonella enterica* serovar Pullorum, which can spread through both horizontal and vertical transmissions. To eradicate the pullorum disease from poultry farms, it is necessary to specifically monitor the prevalence of the bacterial infection in adult chicks. In this study, we constructed a new competitive ELISA method based on the development of monoclonal antibodies (MAbs) against a specific immunogen of *S*. Pullorum, IpaJ protein. In total, eight MAbs against IpaJ were prepared using the purified recombinant His-IpaJ protein as the immunogen. Characterization of the eight MAbs demonstrated that 4G5 can be used as the competitive antibody in ELISA. A competitive ELISA was subsequently developed using purified MBP-IpaJ as the capture (0.5 μg/ml) and the HRP-labeled 4G5 (0.14 μg/ml) as the competitive antibody, respectively. A specificity test demonstrated that the ELISA assay can differentiate antisera of *S*. Pullorum-infected chickens from that of *S*. Gallinarum and *S*. Enteritidis. Furthermore, 4 out of 200 clinical antisera collected from a poultry farm were detected to be *S*. Pulloram positive using this method. The plate agglutination test (PAT) and the previously established indirect ELISA confirmed that these positive antisera reacted specifically with *S*. Pullorum. We propose that the established competitive ELISA assay based on MAb against IpaJ protein, is a novel and quick method that can detect *S*. Pullroum infection in the poultry industry.

## Introduction

Avian salmonellosis remains a big threat to the poultry industry and the public health system. The pullorum disease has mostly been eradicated in many developed countries in Europe, America, and Asia, apart from sporadic incidences in free-ranging backyards. However, it remains endemic in some developing countries like China (1). Monitoring the prevalence of pullorum diseases or adult chickens harboring the pathogen (*Salmonella enterica* serovar Pullorum, *S*. Pullorum) in poultry farms is necessary to control the transmission of the bacteria for the eradication of the disease. However, the traditional method to diagnose the disease is to identify the pathogen in infected chickens through biochemical tests or molecular detection methods including PCR analysis (2–4). All of these methods need the bacteria to be extracted from the chicken samples, which is time-consuming. Additionally, the adult birds which are the carrier of *S*. Pullorum may continue to shed *Salmonella* in eggs because of its colonization in the reproductive tract, but the pathogen cannot be detected in the intestine, liver, and spleen. Thus, new methods based on the detection of antibodies can improve the efficiency of pathogen detection, as opposed to the traditional methods (5).

Previous studies have developed many serological detection methods to identify the *Salmonella* infection in human or animals. Most of these studies used cell surface proteins as the detection antigens, such as LPS, FliC, and so on (5–8). However, the specificity of these molecules is not able to differentiate the various *Salmonella* serotypes. For example, the plate agglutination test (PAT) based on O9 antigens could not differentiate *S*. Pullorum from *S*. Gallinarum, and *S*. Enteritidis (1). To establish a serological detection method specific to *S*. Pullorum infection, an antigen specific to *S*. Pullorum should be found and evaluated against the antibodies produced during the bacterial infection. Our previous studies demonstrated that IpaJ protein was prevalent in 99.08% of *S*. Pullorum and was deficient in other *Salmonella* serotypes, including the closely related *S*. Gallinarum and *S*. Enteritidis species. Additionally, the antibodies against IpaJ protein could be detected from the first to the twelfth week after *S*. Pullorum infection in chicken sera (9).

Based on our previous established indirect ELISA assay to detect antibodies against IpaJ protein in chicken antisera, we tried to establish a new and efficient method to monitor *S*. Pullorum infection in poultry industry. In the present study, we prepared monoclonal antibodies (MAbs) against IpaJ and developed a competitive ELISA method to detect *S*. Pullorum infection in poultry farms with one of the MAbs.

## Materials and Methods

### Bacterial Strains, Plasmids, and Proteins

*S*. Pullorum reference strain C79-13 was offered by China Institute of Veterinary Drug Control. The prokaryotic expression vector pColdI (Takara, Japan) was used to construct recombinant expression plasmids in host cells *E. coli* BL21(DE3). The bacteria with recombinant plasmids were grown in Luria Bertani (LB) broth with Ampicillin (100 μg/ml). The purified MBP-IpaJ was collected and preserved in our laboratory (9).

### Construction of Recombinant Expression Plasmid

According to the published *ipaJ* gene sequence (Accession Number: GU949535) in GenBank, forward and reverse primers pColdI-*ipaJ*-F/R (coldI-*ipaJ*-F: CATCATCATCATCATCATATGCGGTTAAAATTTATCAG; coldI-*ipaJ*-R: TCTAGACTGCAGGTCGACTCAAGCTGACAAGACAATAGA) were designed to clone the gene from C79-13 using PCR. PCR analysis was performed in a 50 μ reaction system: 2.5 U of Taq DNA polymerase, 10 μ 5 × SF buffer, 200 μM dNTP mix, 0.4 μM upstream and downstream primers, and 1 μl of DNA template. The amplification procedure was started with a pre-denaturation at 95°C for 5 min, followed by 30 cycles of 95°C for 50 s, 61°C for 50 s, and 72°C for 1 min, and terminated with a final extension at 72°C for 10 min. Next, the PCR products were resolved on a 1% agarose gel and purified using MiniBEST Agarose Gel DNA Extraction kit (Takara, Japan). The purified PCR products of *ipaJ* were then ligated to pColdI vector and transformed into the competent *E. coli* DH5α. The positive colonies carrying the recombinant plasmid were identified using PCR and sequencing analysis. The recombinant plasmids pColdI-*ipaJ* were then transformed into *E. coli* BL21(DE3) competent cells to produce BL21(DE3)-pColdI-*ipaJ*.

### Expression and Purification of the Recombinant His-IpaJ

An overnight culture of recombinant bacteria BL21(DE3)-pColdI-*ipaJ* was inoculated into fresh LB medium with ampicillin at 1:100 dilution. When the OD600 was between 0.4 and 0.6, the inducer IPTG was added to the medium with the final concentration of 0.5 mM to induce protein expression. The bacteria were then cultured at 15°C for 24 h with continuous shaking at 150–180 rpm. The bacterial pellets were collected for ultrasonic lysis, and the precipitate including His-IpaJ protein as inclusive body was subjected to SDS-PAGE followed by purification from the gel according to the protocol of “Purification proteins from polyacrylamide gels (TECH TIP #51, Thermo scientific, USA)” with a few modifications. Briefly, gel was stained with 1 M KCl for 3–5 min, the white stained protein band of interest was excised and cut into pieces by a clean pestle. The excised gel pieces were kept in a clean microcentrifuge tube and 0.5–1 ml of elution buffer (50 mM Tris-HCl, 150 mM NaCl, and 0.1 mM EDTA; pH7.5) was added to completely immerse the gel pieces; After overnight incubation at 30°C in a rotary shaker, the tube was centrifuged at 5,000–10,000 × g for 10 min and the supernatant was pipetted into a new microcentrifuge tube. The supernatant was tested by SDS-PAGE for purified protein, and the concentration of the protein was monitored by Quick Start^TM^ Bradford protein assay (Bio-rad, USA).

### Immunization of Mice With His-IpaJ

To prepare the B cells producing antibodies against IpaJ protein, 6–8 weeks old BALB/c mice were immunized with His-IpaJ protein (100 μg per mouse) by intraperitoneal (i.p.) injection. The immunization was performed twice every 2 weeks. Three days prior to fusion, 100 μg of His-IpaJ was intravenously injected into the mice. The spleen cells were then fused with sp2/0 cells by using the lymphocyte hybridoma technique (10). All of the animal experiments and managements were undertaken by the permission of the Animal Welfare and Ethics Committees of Yangzhou University, and complied with the guidelines of the institutional administrative committee and ethics committee of laboratory animals.

### Preparation of Monoclonal Antibodies Against IpaJ

The positive hybridoma clones expressing antibodies against His-IpaJ were screened using the previously established indirect ELISA method, with MBP-IpaJ recombinant protein as the coating antigen (9). Positive clones were sub-cloned three times by the limiting dilution method. The Ig sub-class of MAbs were identified by using a mouse mAb isotyping kit (Sigma, USA) according to the manufacturer's instruction. The positive hybridoma cell lines secreting anti-IpaJ MAbs were injected intraperitoneally into BALB/c mice to grow and proliferate. Ascites fluids containing abundant anti-IpaJ MAbs were collected from the immunized mice and purified by protein A chromatography (GE Healthcare). The purified MAbs were send to GenScript Biotechnique Company (Nanjing, China) for biotinylation with HRP.

### Western Blot Analysis

The cell lysates or purified recombinant proteins were subjected to SDS-PAGE on a 12% polyacrylamide gel in Tris-glycine running buffer (pH 8.7), and transferred to PVDF membrane (Pall, USA) by Pyxis^TM^ Gel Processor (Pyxis, China). The PVDF membrane was blocked in 5% BSA and then incubated with anti-MBP antibody or anti-IpaJ MAbs in blocking buffer for 2 h at 37°C. After washing, the membranes were incubated with goat anti-mouse IgG-HRP (Sigma, USA) for 1 h. The PVDF membrane was then stained with the ECL chromogenic kit (Thermo, USA) and scanned using the Amersham Imager 600 imagers (GE Healthcare, USA).

### Construction of a Competitive ELISA Assay

To determine the best working concentration of the coating antigen and the MAb in the competitive ELISA assay, 96-well plates were coated with the purified protein MBP-IpaJ at the following concentrations: 4, 2, 1, 0.5, and 0.25 μg/ml. Twelve wells per concentration were coated with 100 μl per well at 4°C for 14–16 h. Subsequently, the plates were washed three times and then blocked with PBS containing 2% BSA in PBS at 37°C for 2 h. After washing, 50 μl of the doubling diluted HPR-labeled MAb from 1:1,000 to 1:64,000 and 50 μl of the doubling diluted *S*. Pullorum positive antiserum from 1:10 to 1:10,240 were mixed and added into each cell and incubated for 2 h at 37°C. The wells were then washed as above. HPR activity was revealed by adding 3,3′, 5,5′-Tetramethylbenzidine (TMB) at 37°C and incubating for 10 min, and then stopped by adding 2 M H_2_SO_4_. The OD450 was measured to determine the best concentration of the MBP-IpaJ and the MAb used in the competitive ELISA.

Based on the working concentration of the coating protein and the MAb, the dilution ratio of the detected antiserum was determined as follows: 50 μl of the *S*. Pullorum positive antiserum was double diluted from 1:2 to 1:128 and 50 μl of the HRP-labeled MAb was added into the ELISA plate coated with the MBP-IpaJ. The OD450 was measured to calculate the inhibition rate of the antiserum to the MAb.

### Determination of Cut-Off Value for the Competitive ELISA Assay

The specificity and sensitivity of the competitive ELISA assay were checked using other antisera from chickens infected with *S*. Pullorum or other avian *Salmonella* serotypes such as *S*. Gallinarum, *S*. Enteritidis, *S*. Typhimurium, and other poultry bacteria, including *E. coli*, and *Shigella flexneri*. The antisera from chickens infected with these bacteria were subjected to the competitive ELISA assay to evaluate the specificity and determine the cut-off value on the basis of the average control samples. The Gradprism 6.0 software was used to analyze the data and produce ROC curve for determination of the cut-off value.

### Application of the Competitive ELISA Assay to Clinical Samples

In a poultry farm, 200 clinical antiserum samples were collected from chickens. The samples were preserved at −70°C and subsequently monitored using the established competitive ELISA assay. An assay for each sample was performed in triplicates, and the detected positive samples were confirmed by PAT and the indirect ELISA assay (9).

## Results

### Expression and Purification of Recombination Protein His-IpaJ

**Figure 1:**
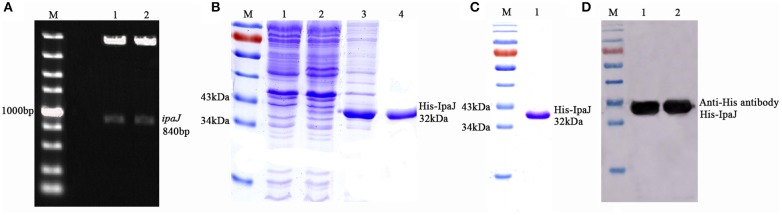
Expression, purification, and identification of recombinant expressed protein His-IpaJ. **(A)** Construction of the prokaryotic recombinant plasmid pColdI-*ipaJ*. Lane 1,2: plasmid digested with *Nde*I and *Sal*I (Takara, Japan). **(B)** SDS-PAGE analysis of expressed recombinant protein His-IpaJ. Lane 1: induced BL21(DE3)-pColdI as negative control; non-induced BL21(DE3)-pColdI-*ipaJ*. Lane 3: the supernatants of induced BL21(DE3)-pColdI-*ipaJ*. Lane 4: the precipitants of induced BL21(DE3)-pColdI-*ipaJ*. **(C)** Purified recombinant His-IpaJ protein (32 KDa). **(D)** Western-blot analysis of the recombinant His-IpaJ protein, using antibody against His Tag.

The *ipaJ* gene of 840 bp was successfully amplified from C79-13 and cloned into the prokaryotic vector pColdI, to produce pColdI-*ipaJ*. The sequence of *ipaJ* was identified using sequencing analysis and digestion with restriction enzymes (Figure 1A).

The recombinant bacteria BL21(DE3)-pColdI-*ipaJ* was induced by 0.5 mM IPTG, and the SDS-PAGE results confirmed that His-IpaJ was successfully expressed in the host cells (Figure 1B). The recombinant protein was estimated to be approximately 32 KDa in the form of inclusion body in BL21(DE3). The His-IpaJ protein was then purified from polyacrylamide gels (Figure 1C).

The His-IpaJ protein expressed and purified from BL21(DE3) was further identified by Western blot analysis with anti-His antibody. The results showed that the expressed recombinant protein His-IpaJ could specifically react with anti-His antibody (Figure 1D).

### Generation and Characterization of MAbs Against His-IpaJ

**Figure 2:**
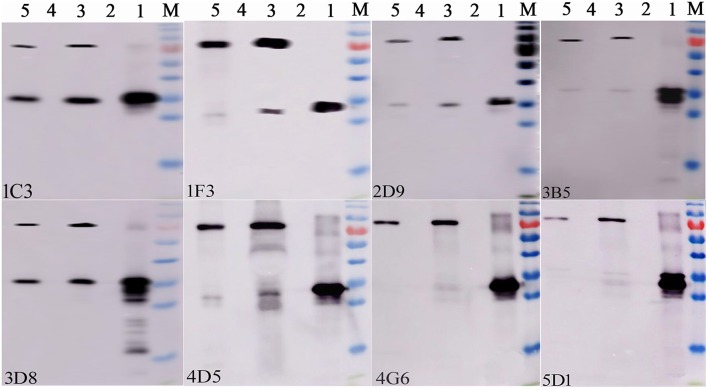
Identification of the reactive specificity of monoclonal antibodies against IpaJ protein by Western blot. Lane 1: Purified His-IpaJ protein (32 KDa); Lane 2: the supernatants of induced BL21(DE3)-pColdI; Lane 3: Purified MBP-IpaJ protein (71 KDa); Lane 4: the supernatants of induced BL21(DE3)-pMAL-c5X. Lane 5: Purified MBP-IpaJ (from *Shigella flexneri*) protein. The 1C3, 1F3, 2D5, 3B5, 3D8, 4D5, 4G6, and 5D1 represents different monoclonal hybridoma cell lines.

**Figure 3:**
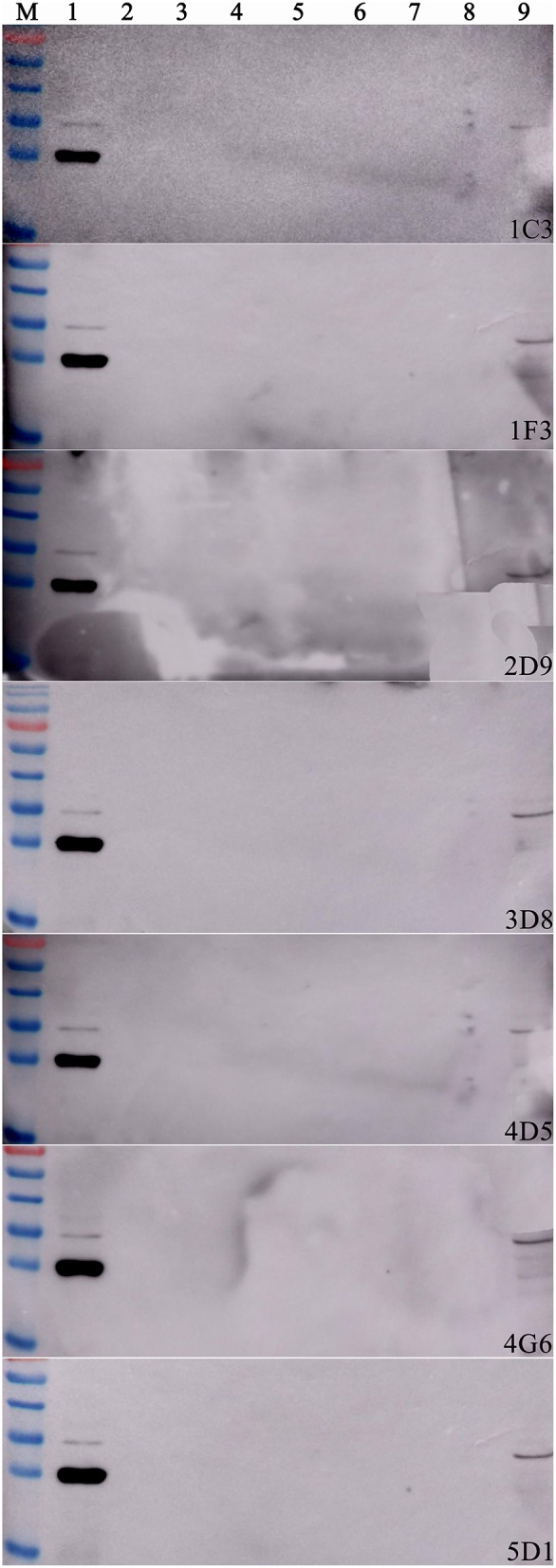
The reactive specificity of monoclonal antibodies against proteins expressed by different *Salmonella* serotypes. The supernatants of different Salmonella serotypes were used as the antigens. *S*. Pullorum C79-13 strain was used as a positive control (Lane 1); *S*. Gallinarum Sg9 (Lane 2); *S*. Enteritidis P125109 (Lane 3); *S*. Dublin D2 (Lane 4); *S*. Typhimurium SL1344 (Lane 5); *S*. Derby LQSD15 (Lane 6); *S*. Indiana LQSI12 (Lane 7); *Escherichia coli* BL21(DE3) was used as a negative control (Lane 8); *Shigella flexneri* SNI carrying IpaJ was used as another positive control.

After two independent fusions and three sub-clonings, eight hybridomas (1C3, 1F3, 2D9, 3B5, 3D8, 4D5, and 5D1) were obtained and selected for further studies based on their strong reactivity with MBP-IpaJ. The results of IgG isotypes showed that four MAbs (1C3, 2D9, 4G6, and 5D1) were IgG1, two MAbs (1F3 and 3D8) were IgG2b and two MAbs (3B5 and 4D5) were IgM (Table 1). The titers of culture supernatants and ascites of each MAb revealed that 4G6 and 5D1 were better than the other MAbs (Table 1). Western blot analysis confirmed that all of the eight MAbs could react specifically with the His-IpaJ and the MBP-IpaJ (Figure 2). Additionally, seven MAbs showed strong reaction with IpaJ secreted by *S*. Pullorum cultured in LB medium by Western blot analysis (Figure 3), but did not react with the lysates of other *Salmonella* serotypes or *E. coli*. However, the Western blot analysis revealed that these MAbs also reacted with supernatants of *Shigella flexneria*, since *Shigella flexneria* expressed a similar IpaJ protein, with 49% homology to the IpaJ in *S*. Pullorum.

**Table 1:** Characteristics of eight monoclonal antibodies against IpaJ.

**Hybridoma**	**Antibody types**	**Titers (culture supernatant)**	**Titers (ascites)**
1C3	IgG1	200	2,000
1F3	IgG2b	1,600	256,000
2D9	IgG1	200	1,000
3B5	IgM	200	4,000
3D8	IgG2b	1,600	128,000
4D5	IgM	6,400	128,000
4G6	IgG1	6,400	2,048,000
5D1	IgG1	6,400	4,096,000

### The Establishment of a Competitive ELISA Assay for *S*. Pullorum Infection

**Figure 4:**
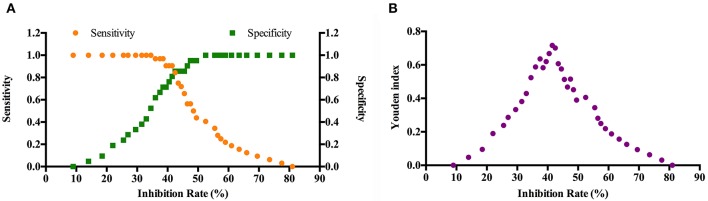
Determination of the cut-off value and Youden index of the competitive ELISA method. Thirty two positive antisera from *S*. Pullorum-infected chickens and 25 negative antisera from SPF chickens not exposed to *Salmonella* were subjected to the ELISA assay to obtain the cut-off value **(A)** and the Youden index **(B)**. The cut-off value of 40.5 represents when the inhibition rate of antisera samples should be ≥40.5.

Our previous study showed that chickens could produce antibodies against IpaJ protein 1 week after infection with *S*. Pullorum and kept at a stable level for up to 12 weeks (9). In order to detect *S*. Pullorum infection, a competitive ELISA was developed using purified MBP-IpaJ as capture and HRP-labeled MAb 4G5 as detection antibodies, respectively. The checkerboard analysis revealed that the appropriate concentration of the coating antigen was 0.5 μg/ml, and the working dilution of the HRP-4G5 was 0.14 μg/ml (Table 2). The inhibition rate detected by positive antisera from *S*. Pullorum infected chickens demonstrated that the working dilution ratio of antisera was 1:16 (Supplementary Figure 1). The cross-link point between sensitivity and specificity curves reflected that the cut-off for inhibition rate was 40.5, and the Youden index was 0.668 (Figure 4). This demonstrated that when the inhibition rate was ≥40.5, the antiserum samples were considered positive for antibodies against IpaJ, and vice versa.

**Table 2:** Checker-board analysis for concentration of coating protein (MBP-IpaJ) and antibody (HRP-4G6) in the competitive ELISA method.

**MBP-IpaJ (μg/ml)**	**HRP-4G6 (μg/ml)**						
	2.24	1.12	0.56	0.28	**0.14**	0.07	0.035
0.25	65.89^a^	62.05	62.78	57.67	53.61	49.49	35.60
**0.50**	47.25	57.58	63.47	64.10	**67.12**^b^	56.65	48.87
1.00	62.51	53.73	50.61	52.21	52.80	50.76	49.14
2.00	51.42	56.72	59.84	50.46	55.89	58.51	54.40
4.00	50.77	51.14	60.59	56.94	30.76	50.51	48.87

a *represents the inhibition rate (%) of ELISA assay*.

b *represents when the inhibition rate reached 67.12%, the 0.14 μg/ml of HRP-4G6 and 0.5 μg/ml of MBP-IpaJ was used in the established competitive ELISA assay as the concentration of antibody and coating protein, respectively*.

### Application of the Competitive ELISA Method to Monitor Clinical Samples

The competitive ELISA method was used to detect 200 clinical antiserum samples from a poultry farm, and the result showed that four samples were positive for antibodies against IpaJ. The PAT method and the indirect ELISA assay further confirmed that all of the four antiserum samples could react with *S*. Pullorum C79-13. However, the PAT cannot discriminate antisera from chickens infected by *S*. Pullorum, *S*. Gallinarum, or *S*. Enteritidis with the same O9 antigen, and the indirect ELISA method is time-consuming compared to the competitive ELISA assay.

## Discussion

*S*. Pullorum and the other avian pathogens, *S*. Enteritidis and *S*. Gallinarum, are group D members in the well-recognized Kauffmann-White *Salmonella* classification scheme (11). Since these serotypes have similar LPS O-antigen structure, the established ELISA methods based on the anti-LPS MAbs can be used to monitor avian salmonellosis caused by *S*. Pullorum, *S*. Gallinarum, *S*. Enteritidis, and other *Salmonella* group D serotypes (12). These methods can be applied for the surveillance of avian salmonellosis in the poultry farms, but cannot identify the exact serotype of the pathogen. In order to differentiate *S*. Pullorum-infection from other serotype infections, we established a competitive ELISA method to specifically detect *S*. Pullorum-infected chickens.

IpaJ has been identified as an immunogen, expressed specifically by >99% of *S*. Pullorum isolates, and not detected in other closely related group D serotypes including *S*. Gallinarum, and *S*. Enteritidis (4). Our previous study demonstrated that the protein could induce antibody production at 1 week post-infection and persist for over 12 weeks (9). An indirect ELISA method based on the MBP-IpaJ recombinant protein as the coating protein was established and applied for detection of antibodies from *S*. Pullorum-infected chickens (9). However, since the IpaJ protein has the MBP tag, the indirect ELISA method has low specificity. Therefore, the MAbs against IpaJ were obtained and the competitive ELISA method was constructed and applied to the clinical samples in this study. The antiserum samples and the HRP-labeled MAbs were added at the same time in the competitive ELISA assay, which saved more time than the indirect ELISA method. In *S*. Pullorum, the IpaJ works as an immune regulator involved in inhibiting T-cell immune response (13). However, the molecular mechanism behind its immune modulatory role remains unknown. Thus, the MAbs against the IpaJ identified in this study may also be used as a tool to further explore its function.

We conclude, that the present study obtained eight MAbs against the IpaJ of *S*. Pullorum, and developed a competitive ELISA method for identifying *S*. Pullorum infection in poultry farms based on the MAb 4G5. The method showed enhanced sensitivity and specificity compared to our previously established indirect ELISA method. We thus propose that this competitive ELISA is a quick and efficient method that can be applied for the surveillance of *S*. Pullorum infection in the poultry industry.

## Data Availability Statement

All datasets generated for this study are included in the article/Supplementary Material.

## Ethics Statement

The animal study was reviewed and approved by the Animal Welfare and Ethics Committees of Yangzhou University.

## Author Contributions

KY, QL, and XJ designed and coordinated the study. KY, JR, and YZ performed the experiments and wrote the manuscript. KY, LX, CY, YL, and YY analyzed the data. QL and XJ reviewed and edited the manuscript.

### Conflict of Interest

The authors declare that the research was conducted in the absence of any commercial or financial relationships that could be construed as a potential conflict of interest.
