# Supplementary material for: Application of Monoclonal Antibodies Developed Against the IpaJ Protein for Detection of Chickens Infected With Salmonella enterica Serovar Pullorum Using Competitive ELISA
Source: Front Vet Sci. 2019 Nov 5;6:386. doi: 10.3389/fvets.2019.00386 (PMC6848452; doi:10.3389/fvets.2019.00386)
Supplement: Supplementary file 1 [file Data_Sheet_1.PDF]

Supplementary Material

Application of monoclonal antibodies developed against the IpaJ protein for detection of chickens infected with *Salmonella enterica* serovar Pullorum using competitive ELISA

Kequan Yin [1,2,3,a], Jingwei Ren [1,2,3,a], Yue Zhu [1,2,3,a], Lijuan Xu [1,2,3], Chao Yin [1,2,3], Yang Li [1,2,3], Yu Yuan [1,2,3], Qiuchun Li [1,2,3,] *, Xinan Jiao [1,2,3]

*** Correspondence:**

Dr. Qiuchun Li, E-mail:qcli@yzu.edu.cn

Supplementary Figure

For more information on Supplementary Material and for details on the different file types accepted, please see here.

Figure legends

Supplementary Figure S1.

The working concentration of the antisera used in the competitive ELISA assay. The positive antisera from *S.* Pullorum infected chickens were diluted serially from 1:2 to 1:128, and subjected to the competitive ELSIA assay. The inhibition rate was ~65% at the 1:16 dilution point, which was then used as the working concentration for antisera samples in the ELISA assay.
