# Supplementary figures and images for: Application of Monoclonal Antibodies Developed Against the IpaJ Protein for Detection of Chickens Infected With Salmonella enterica Serovar Pullorum Using Competitive ELISA
Source: Front Vet Sci. 2019 Nov 5;6:386. doi: 10.3389/fvets.2019.00386 (PMC6848452; doi:10.3389/fvets.2019.00386)

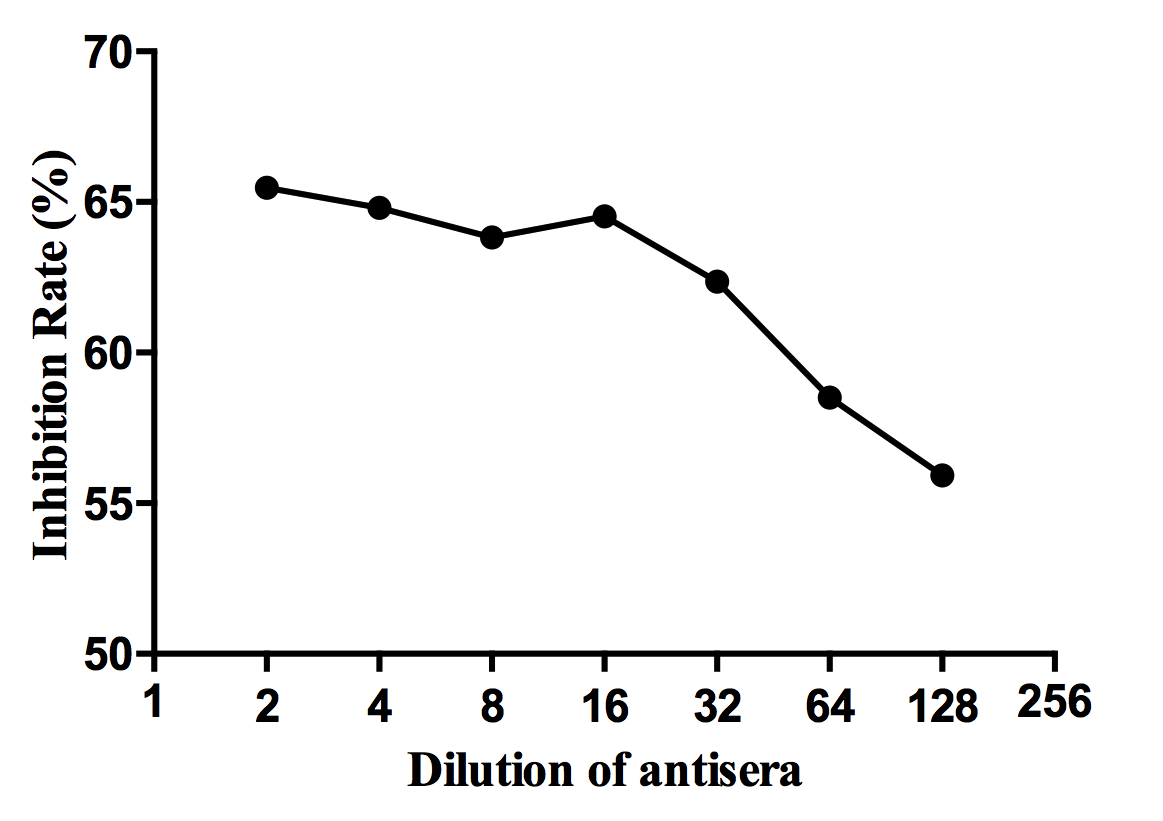

Supplement: Supplementary file 2 [file Image_1.TIFF]
